# Association between precipitation and mortality due to diarrheal diseases by climate zone: A multi-country modeling study

**DOI:** 10.1097/EE9.0000000000000320

**Published:** 2024-07-17

**Authors:** Paul L. C. Chua, Aurelio Tobias, Lina Madaniyazi, Chris Fook Sheng Ng, Vera Ling Hui Phung, Sze Hang Fu, Peter S. Rodriguez, Patrick Brown, Micheline de Sousa Zanotti Stagliorio Coelho, Paulo Hilario Nascimento Saldiva, Noah Scovronick, Aniruddha Deshpande, Miguel Antonio S. Salazar, Miguel Manuel C. Dorotan, Kraichat Tantrakarnapa, Wissanupong Kliengchuay, Rosana Abrutzky, Gabriel Carrasco-Escobar, Dominic Roye, Simon Hales, Masahiro Hashizume

**Affiliations:** a Department of Global Health Policy, Graduate School of Medicine, University of Tokyo, Bunkyo-ku, Tokyo, Japan; b Institute of Environmental Assessment and Water Research, Spanish Council for Scientific Research, Barcelona, Spain; c Department of Global Health, School of Tropical Medicine and Global Health, Nagasaki University, Nagasaki, Japan; d Centre for Global Health Research, St. Michael’s Hospital and University of Toronto, Toronto, Ontario, Canada; e Department of Statistical Sciences, University of Toronto, Toronto, Ontario, Canada; f Department of Pathology, Faculty of Medicine, University of São Paulo, São Paulo, Brazil; g Department of Environmental Health, Rollins School of Public Health, Emory University, Atlanta, Georgia; h Department of Epidemiology, Rollins School of Public Health, Emory University, Atlanta, Georgia; i Alliance for Improving Health Outcomes, Inc., Quezon City, Philippines; j Department of Social and Environmental Medicine, Faculty of Tropical Medicine, Mahidol University, Ratchathewi, Bangkok, Thailand; k Instituto de Investigaciones Gino Germani, Facultad de Ciencias Sociales, Universidad de Buenos Aires, Ciudad Autónoma de Buenos Aires, Argentina; l Scripps Institution of Oceanography, University of California, San Diego, California; m Health Innovation Laboratory, Institute of Tropical Medicine “Alexander von Humboldt,” Universidad Peruana Cayetano Heredia, Lima, Peru; n Climate Research Foundation, Madrid, Spain; o Department of Public Health, University of Otago, Newtown, Wellington, New Zealand

**Keywords:** Precipitation, Diarrheal mortality, Poisson regression models, Meta-analysis

## Abstract

**Background:**

Precipitation could affect the transmission of diarrheal diseases. The diverse precipitation patterns across different climates might influence the degree of diarrheal risk from precipitation. This study determined the associations between precipitation and diarrheal mortality in tropical, temperate, and arid climate regions.

**Methods:**

Daily counts of diarrheal mortality and 28-day cumulative precipitation from 1997 to 2019 were analyzed across 29 locations in eight middle-income countries (Argentina, Brazil, Costa Rica, India, Peru, the Philippines, South Africa, and Thailand). A two-stage approach was employed: the first stage is conditional Poisson regression models for each location, and the second stage is meta-analysis for pooling location-specific coefficients by climate zone.

**Results:**

In tropical climates, higher precipitation increases the risk of diarrheal mortality. Under extremely wet conditions (95th percentile of 28-day cumulative precipitation), diarrheal mortality increased by 17.8% (95% confidence interval [CI] = 10.4%, 25.7%) compared with minimum-risk precipitation. For temperate and arid climates, diarrheal mortality increases in both dry and wet conditions. In extremely dry conditions (fifth percentile of 28-day cumulative precipitation), diarrheal mortality risk increases by 3.8% (95% CI = 1.2%, 6.5%) for temperate and 5.5% (95% CI = 1.0%, 10.2%) for arid climates. Similarly, under extremely wet conditions, diarrheal mortality risk increases by 2.5% (95% CI = −0.1%, 5.1%) for temperate and 4.1% (95% CI = 1.1%, 7.3%) for arid climates.

**Conclusions:**

Associations between precipitation and diarrheal mortality exhibit variations across different climate zones. It is crucial to consider climate-specific variations when generating global projections of future precipitation-related diarrheal mortality.

**What this study adds**

Previous studies have analyzed the impact of precipitation on diarrhea morbidity. Given that mortality due to diarrheal diseases is a global health concern, this study, for the first time, investigated its association with precipitation using a multi-country dataset. Distinct differences were observed in the associations between precipitation and mortality across climates and regions. Arid and temperate locations showed a higher mortality risk associated with both extreme precipitation accumulation and deficiency. In tropical locations, mortality risk only increased with precipitation accumulation. The highest mortality risks for precipitation deficiency and accumulation were observed in South American and Asian locations, respectively.

## Introduction

Diarrheal diseases remain a serious global health problem, particularly in low- and middle-income countries (LMICs). In 2019, it was ranked the fifth leading cause of mortality, with an estimated 1.5 million deaths of all ages worldwide.^1^ Major reductions have been made so far, from a mortality rate of 54 deaths per 100,000 in 1990 to 20 deaths per 100,000 in 2019 attributable to improved access to sanitation, child nutrition, delivery of oral rehydration solutions, and rotavirus vaccination.^2,3^ This progress in reducing diarrheal mortality could be threatened in the future by climate change through altering ecologic and human processes, which influence the transmission of enteric pathogens. For example, the future rise in ambient temperatures due to climate change is projected to cause up to 83,888 additional deaths due to diarrheal diseases by the end of the 21st century (2080–2095) compared with a historical baseline (1990–2005).^4^ Higher ambient temperatures are expected to aid the survival and growth of bacterial and protozoal pathogens in the environment or lead to poorer access and availability of water and sanitation.^5^

Apart from ambient temperature, precipitation is another climatic factor that could both directly and indirectly affect the transmission of diarrheal diseases. A primary mode of transmission is through heavy precipitation events that lead to flushing or mobilization of enteric pathogens on environmental surfaces like soils and waters to other areas exposing more individuals to diarrheal diseases.^5^ The mobilization of pathogens, especially in heavy precipitation events, can lead to contamination of drinking water sources. Heavy precipitation events could also contaminate drinking water sources or interrupt water and sanitation services, forcing the use of unsafe water, ingestion of contaminated drinking water, and poor hand hygiene. This relationship between heavy precipitation and diarrheal incidence has been reported by numerous epidemiological studies.^5^ Additionally, low precipitation or dry events can promote the transmission of diarrheal diseases by accumulating or concentrating the enteric pathogens in the environment that could be spread mechanically by wind or flushed by sudden precipitation. Climate change has influenced the increase in number and frequency of both extreme precipitation and dry spells globally, depending on the geographical region,^6,7^ and this could potentially promote the transmission of diarrheal diseases. However, there has been no future projection study of diarrheal excess deaths considering future changes in precipitation patterns driven by climate change. This is partly because of the absence of an epidemiological study examining the association between precipitation and diarrheal mortality at a global scale.

The impact of precipitation on the risk of diarrheal diseases could vary according to climate zones because of their varying precipitation amount and spatiotemporal patterns. Tropical regions received the most precipitation and would generally have wetter conditions compared with the rest of the climate zones.^8^ Temperate regions receive moderate precipitation across the year, and they experience sporadic drought,^9^ while arid regions receive the least amount of precipitation.^8^ A previous study, which utilized diarrheal morbidity data (i.e., caregiver-reported diarrhea episodes among very young children) from Demographic and Health Surveys, reported opposing precipitation–diarrheal mortality associations between tropical and temperate climates.^10^ A limitation in using cross-sectionally ascertained data like that of the Demographic Health Survey (DHS; e.g., every ≈5 years) is the lack of temporal and seasonal characteristics and changes in precipitation and diarrheal diseases. Another limitation is that caregiver-reported diarrhea episodes are not assessed by a medical professional and are subject to recall bias. Using routine data that are regularly collected and analyzed by a medical professional can overcome these limitations.

This study aims to quantify the association between precipitation and mortality due to diarrheal diseases by climate zone. We used vital statistics data from eight middle-income countries to collect diarrheal mortality. To our knowledge, this is the first study of mortality from diarrheal diseases and its association with precipitation for multiple countries in different continents.

## Methods

### Data sources

**Figure 1.**
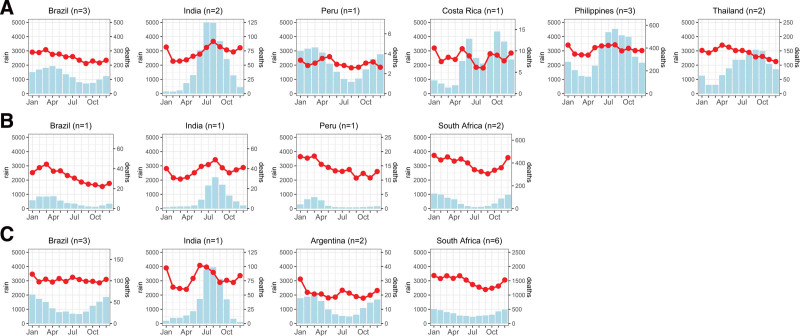
Monthly averages of total diarrheal deaths and 28-day cumulative precipitation by climate and country. Climate A, B, and C refer to tropical, arid, and temperate, respectively. Red circles refer to diarrheal deaths, and blue bars refer to 28-day cumulative (running sums) precipitation in millimeters. n refers to the number of included within-country climatic regions.

Deaths coded with the Tenth Revision of the International Classification of Diseases (ICD-10) A00–A09, which are intestinal infectious diseases, were collected from the national statistics offices of Argentina, Brazil, Costa Rica, Peru, the Philippines, South Africa, and Thailand. For India, deaths coded with ICD-10 A09 were from the Million Death Study, which was a nationally representative survey undertaken by the Registrar General of India’s Sample Registration System.^11^ The data include the location of death in various administrative levels: province level for Argentina and Peru, district level for South Africa and Thailand, municipality level for Brazil and the Philippines, and village/PIN code level for India (Supplemental Document, Figure 1; http://links.lww.com/EE/A289). The data are from 1997 to 2019 but the number of years varies by country (e.g., South Africa has 1997–2013 while Costa Rica has 2014–2019). Additional details of the mortality data are presented in Supplemental Document, Table 1; http://links.lww.com/EE/A289.

The data for climate zone, weather variables, population density, gross domestic product (GDP), and water and sanitation indicators were collected from open data sources (Supplemental Document, Tables 2 and 3; http://links.lww.com/EE/A289). Climate zone classification was based on the Köppen-Geiger system, wherein 30 sub-climate classifications are categorized according to several temperature and precipitation criteria,^8^ and was extracted from the raster developed by Beck et al.^12^ Hourly total precipitation and 2-m air temperatures were from the European Centre for Medium-Range Weather Forecasts climate re-analysis 5th generation land component dataset made available by the European Centre for Medium-Range Weather Forecasts.^13^ The total precipitation is the accumulated rain and snow that fall on the Earth’s surface. Human population was from the Gridded Population of the World, version 4.11, dataset provided by the National Aeronautics and Space Administration Socioeconomic Data and Applications Centre and Centre for International Earth Science Information Network.^14^ GDP per capita was from the study by Kummu et al.^15^ For water and sanitation indicators, we used the Institute of Health Metrics and Evaluation gridded dataset for LMICs on the proportion of household with improved sanitation, practicing open defecation, with access to improved drinking water sources, and with piped water.^16^ Argentina was not included in the previous dataset, so we collected the same water and sanitation indicators from the United Nations Children’s fund’s country-level dataset.

### Data aggregation

**Figure 2.**
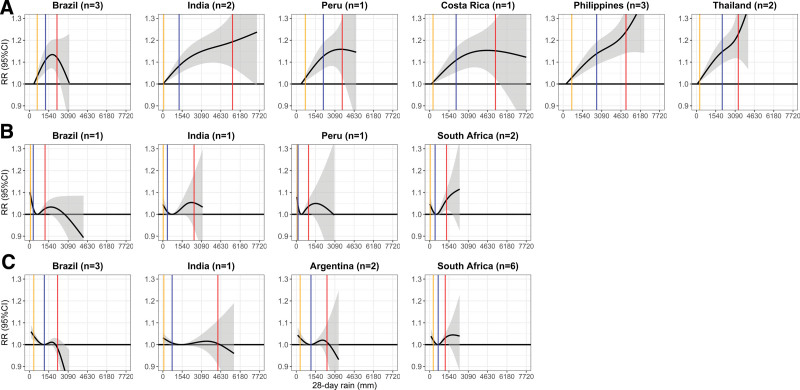
Associations between precipitation and diarrheal mortality by climate–country. Climate A, B, and C refer to tropical, arid, and temperate, respectively. The *y* axis is relative risk (RR) and its 95% CI, and the *x* axis is 28-day cumulative (running sums) precipitation in millimeters. Black lines and gray areas are the RR means and 95% CI, respectively. Orange, blue, and red vertical lines are 5th, 50th, and 95th precipitation percentiles, respectively. n refers to the number of included within-country climatic regions.

We aggregated the diarrheal deaths, total precipitation, and temperatures by day and by within-country climatic region, which is aggregated subnational units with the same climate zone.^17^ First, we computed the sum of deaths by day and location of death at the smallest administrative level or subnational unit. Second, we computed population-weighted averages of daily total precipitation (mm) and temperatures (°C) for each subnational unit adjusting for the local time. Third, we categorized each subnational unit with a sub-climate zone (i.e., third-level climate classification) based on the grids with the greatest total sum of population (i.e., the highest total population counts of sub-climate zone grids within the subnational unit). Lastly, we computed the overall sum of daily diarrheal deaths and average daily weather variables (using diarrheal deaths as weights) by within-country climatic region, by aggregating subnational units with the same sub-climate zone. We limited the time series up to 2019 because patterns of diarrheal deaths changed from 2020 onward. Twenty-nine within-country climatic regions with at least one diarrheal death each month per year were included in the analysis (Supplemental Document, Figure 2; http://links.lww.com/EE/A289). Twenty-eight within-country climatic regions were excluded due to low numbers of deaths over long time periods that could yield highly uncertain results. We used the same approach to extract population density, GDP per capita, and water and sanitation indicators. A detailed description of the data processing and a summary of the included and excluded within-country climatic regions are presented in Supplemental Document, Table 4; http://links.lww.com/EE/A289.

### Statistical analysis

A two-stage approach^18^ was applied to model the association between precipitation and diarrheal mortality by first-level climate zone of tropical, arid, and temperate regions. In the first stage, we modeled the precipitation–diarrheal mortality associations for each within-country climatic region using a time-stratified case-crossover study design, which is an alternative to time series regression to estimate short-term associations of environmental exposures.^19^ We defined the time stratum as the same day of the week (i.e., Monday to Sunday) within the same quarterly months (i.e., January to March, April to June, July to September, and October to December) and year. The selected time stratification provided an approximate ratio of 1 case to 11 controls and was sufficient to adjust for seasonality and trend. The second stage involved pooling within-country climatic region coefficients using meta-analysis to obtain the overall associations for each main climate zone.

In the first stage, we used a conditional quasi-Poisson model^20^ separately for each of the 29 within-country climatic regions:


$$\begin{array}{ll} & \ln\left[E\left(Y_{t,c}\right)\right]=\alpha_{c}+f\left(rain_{t};\beta \right)+s\left(temp_{t,l};\theta \right)\;+I\left(month;\gamma \right), \end{array}$$ (Equation 1)

where E(Y) is the expected daily diarrheal deaths on day t in stratum c, which is year, quarterly months, and day of week (e.g., 2006; January to March; Tuesday); α is the intercept in stratum c; f is the natural cubic B spline of 28-day running sums of precipitation; s is the bidimensional spline function of exposure–lag–response for daily temperature and lag l; I is the indicator function for month; and β, θ, and γ are the parameters of estimation.

We used 28-day running sums of daily total precipitation, or cumulative precipitation, to consider the broad diarrheal disease transmission processes.^5,21^ Deaths that occurred on day $t_{0}$ were matched with prior 28-day precipitation sum $t_{\left[-29,\;\;-1\right]}$. The strategy is based on the understanding that both extremely dry and wet conditions could concurrently spread more pathogens in the environment and negatively affect water, sanitation, and hygiene conditions, leading to serious diarrheal outcomes within the period of a month.^21,22^ We defined extremely dry and wet conditions as the 5th and 95th percentiles of 28-day cumulative precipitation, respectively. The selection of knots at the 50th and 90th percentiles of precipitation was based on prior knowledge of increased risk in dry and wet conditions and the right-skewed distribution of precipitation. We added an indicator of month in the model to account for possible monthly mortality variations in each location.

To adjust for temperature, we used a natural cubic B spline with two knots at the 33rd and 67th percentiles to accommodate nonlinear associations of low and high temperatures.^23^ The lag–response function was modeled using a natural cubic B spline for 0–28 days of lag with an intercept and three internal knots placed at log scale.^23,24^

In the second stage, we used a multilevel meta-regression with restricted maximum likelihood estimation^25^ to combine the β-coefficients representing precipitation–diarrheal mortality associations:


$$\begin{array}{l} \beta_{i,j}=X_{i,j}\gamma +Z_{j}g_{j}, \end{array}$$ (Equation 2)

where β is the coefficient for within-country climatic region i and country j; X is the matrix of fixed-effect predictor for climate zone; and Z is a random term for country with a random coefficient g.

We estimated the overall precipitation–diarrheal mortality associations for each climate zone of tropical, arid, and temperate regions using the meta-regression model. Residual heterogeneity was measured using Cochran’s *Q* test and *I*^2^ statistic. We measured the significance of climate zone as a meta-predictor using the Wald test.^26^ We determined the minimum risk precipitation as the amount of precipitation with the lowest mortality risk restricted between the minimum precipitation and the 90th percentile of the precipitation distribution. We computed the relative risks at the 5th and 95th percentiles of precipitation distributions compared with the minimum risk precipitation to measure the associations in extremely dry and wet conditions, respectively. To obtain country-specific results, we derived the best linear unbiased prediction estimates of the overall precipitation–diarrheal mortality associations. The associations predicted with best linear unbiased prediction provide weighted country–climate estimates with reduced imprecision because it allows locations with small mortality counts to borrow information from those with larger mortality counts.^27^

Sensitivity analyses were performed to assess the robustness of the results by repeating specific analyses with different parameters and model specifications: (1) changing the time stratification of quarterly months by starting from February to April and from March to May onward; (2) changing the time stratification using the same year, month, and day of the week (and removing the month indicator); (3) using shorter running sums of precipitation to 7, 14, and 21 days; (4) removing the month indicator; and (5) adding population density, GDP per capita, and water and sanitation indicators as meta-predictors one at a time to the base meta-regression model in the second stage.

All data processing and analyses were performed using R software for statistical computing (version 4.3.1). The R packages used are listed in Supplemental Document, Table 5; http://links.lww.com/EE/A289.

### Ethical considerations

No research ethics approval was required for the study protocol because the secondary diarrheal mortality data were deidentified (i.e., no names and residential addresses) and aggregated at daily and subnational units. The rest of the secondary data were collected from open data sources. Only the research team had access to all datasets.

## Results

**Figure 3.**
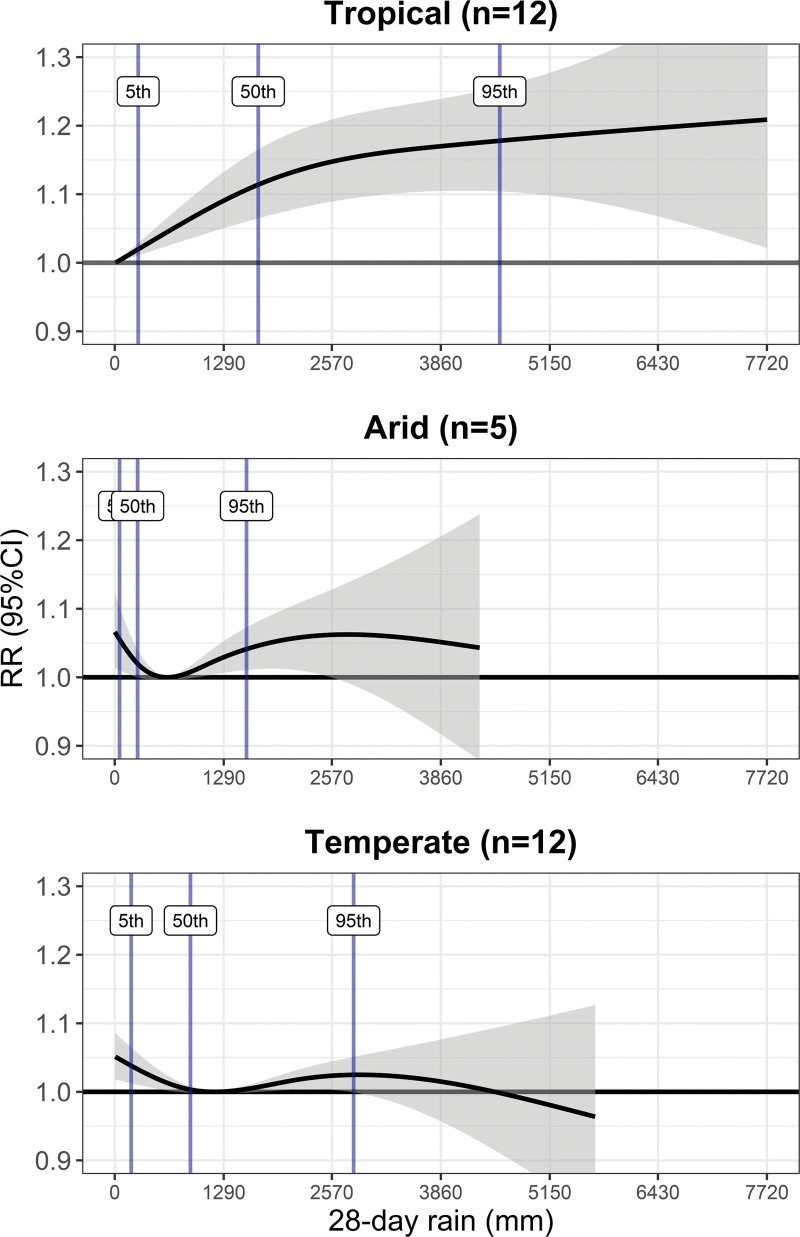
Associations between precipitation and diarrheal mortality by main climate zone. The *y* axis is relative risk (RR) and its 95% CI, and the *x* axis is 28-day cumulative (running sums) precipitation in millimeters. Black lines and gray areas are the RR means and 95% CI, respectively. Blue lines are 5th, 50th, and 95th precipitation percentiles. n refers to the number of included within-country climatic regions.

A total of 591,412 diarrheal deaths were recorded from 1997 to 2019 in the study sites (Supplemental Document, Table 4 and Figure 3; http://links.lww.com/EE/A289). The majority (64% or 380,195 deaths) came from South African locations, followed by Brazil, with 16% (93,825 deaths) of the overall deaths. A low number of deaths were reported in Argentina (2812 deaths), Costa Rica (610 deaths), and Peru (3528 deaths), constituting 1% of the overall diarrheal deaths. Tropical locations, particularly rainforest and monsoon sub-climates, had the highest total precipitation values (Figure 1; Supplemental Document, Figure 4; http://links.lww.com/EE/A289). Arid locations were the driest, with the lowest total precipitation values. Warm mean temperatures were observed in tropical locations, while the rest had colder mean temperatures than tropical locations.

Figure 2 shows the cumulative associations between precipitation and diarrheal mortality by climate and country. For tropical climates, there were differences in the shape of the associations among South American and Asian countries. Brazil, Costa Rica, and Peru’s associations resembled an inverted U shape, which peaked at 86th, 91st, and 93rd percentiles of their respective tropical precipitation distributions (Supplemental Document, Table 6; http://links.lww.com/EE/A289). Asian countries, such as India, the Philippines, and Thailand, had an approximately linear shape, with the risk increasing as precipitation volume increased. For arid climates, all countries had U-shaped associations, but there were slight differences in the risks for dry and wet conditions. Brazil and Peru had a higher risk for dry conditions than wet conditions, while it was the opposite for India and South Africa. For temperate climates, Brazil, India, and Peru’s associations dipped under wet conditions but not for South Africa. For extremely wet conditions (95th percentile precipitation), tropical Philippines had the highest percentage change in precipitation–diarrheal mortality risk (24.0% [95% confidence interval (CI) = 14.1%, 34.7%]), while temperate Brazil had the lowest percentage change (−1.7% [95% CI = −5.9%, 2.8%]). For extremely dry conditions (fifth percentile precipitation), arid Brazil had the highest percentage change (8.7% [95% CI = 3.4%, 14.1%]), while the temperate South Africa had the lowest percentage change (2.0% [95% CI = 0.1%, 3.9%]).

Figure 3 shows the overall cumulative precipitation–diarrheal mortality associations for each climate zone. For tropical climate, the shape of the precipitation–diarrheal mortality association was approximately linear with higher diarrheal risk in wetter conditions and minimum risk precipitation at the lowest precipitation value. Arid and temperate climate exhibited U-shaped associations extending over a large part of the precipitation percentile distribution indicating increased diarrheal risk in both dry and wet conditions. The Table shows the minimum risk precipitation and percentage change of diarrheal mortality risk for extremely dry and wet conditions for each climate zone. In tropical climate, diarrheal mortality increased by 17.8% (95% CI = 10.4%, 25.7%) under extremely wet conditions compared with the minimum risk precipitation. For temperate and arid climates, mortality risk increased in both dry and wet conditions. In extremely dry condition, mortality risk increased by 3.8% (95% CI = 1.2%, 6.5%) for temperate and 5.5% (95% CI = 1.0%, 10.2%) for arid climates. Similarly, under extremely wet conditions, mortality risk increased by 2.5% (95% CI = −0.1%, 5.1%) for temperate and 4.1% (95% CI = 1.1%, 7.3%) for arid climates. The pooled estimates of precipitation–diarrheal mortality associations from the meta-analysis model reported moderate heterogeneity with *I*^2^ of 39% and a significant Cochran’s *Q* test (*P* = 0.0005). Climate had a significant difference in the precipitation–diarrheal mortality associations (*P* < 0.0001).

**Table.** Minimum risk precipitation and percentage change of diarrheal mortality risk at extremely dry and wet conditions by climate zone

Climate	Minimum risk precipitation		Extremely dry (5th percentile precipitation)		Extremely wet (95th percentile precipitation)	
	mm	Percentile	mm	% Change^a^ (95% CI)	mm	% Change^a^ (95% CI)
Tropical^b^	9	0	—	—	4,558	17.8 (10.4, 25.7)
Arid	622	73	57	5.5 (1.0, 10.2)	1,560	4.1 (1.1, 7.3)
Temperate	1,180	64	195	3.8 (1.2, 6.5)	2,828	2.5 (−0.1, 5.1)

a Percentage change relative to minimum risk precipitation.

b Approximately linear in shape of precipitation–diarrheal mortality associations, so fifth percentile values were not included.

Results of sensitivity analysis showed that the shapes of the curves remained largely consistent when changing time stratification, running sum of precipitation days, and month indicator. However, the results for arid and temperate climates exhibited some sensitivity to the February-to-April stratification, same month stratification, and to the exclusion of the month indicator (Supplemental Document, Figures 5–8; http://links.lww.com/EE/A289). For other meta-predictors (or other factors that could explain the associations), only the percentage of households with access to improved sanitation and piped water were significant moderators of precipitation–diarrheal mortality associations (Supplemental Document, Table 7; http://links.lww.com/EE/A289) although none changed the shapes of the risk curves by climate zone (Supplemental Document, Figure 9; http://links.lww.com/EE/A289). The risk for extremely wet conditions was lower when the percentage of improved sanitation and access to piped water were high (Supplemental Document, Figures 10 and 11; http://links.lww.com/EE/A289). On the contrary, the risk for extremely dry conditions was higher given the same high percentage of improved sanitation and access to piped water.

## Discussion

We determined the associations between precipitation and diarrheal mortality and found differences among climate zones in eight middle-income countries. For tropical climate, a positive association with an almost linear shape was found between precipitation and diarrheal mortality with increasing risks under wetter conditions. On the other hand, arid and temperate climates showed U-shaped associations with increased diarrheal mortality risks in both drier and wetter conditions. Our findings are generally in line with the available literature that conceptually presumes the occurrence of increased diarrheal risk in both drier and wetter conditions because these conditions contribute to the concentration and spread of enteric pathogens in the environment.^5,21^

A generally wet environment year-round for tropical regions could influence lesser diarrheal risk possibly because of the dilution of enteric pathogens in the environment.^28^ The DHS study demonstrated this as the authors reported increased diarrheal risk only in drier conditions for locations with tropical climate.^10^ However, a plausible explanation for our opposite findings is the increased contamination of drinking water sources during wetter conditions. Several studies have reported that wetter conditions and heavy precipitation increase the presence of *Escherichia coli* in drinking water from point of collection, which included improved and unimproved water sources, in tropical locations.^29–31^ The consumption of contaminated drinking water would not only increase the chance of enteric infection but could also lead to aggravation of diarrhea severity from ingesting more enteric pathogens and possible coinfections.^32^ This was echoed in our findings wherein higher coverage in piped water access reduced the diarrheal mortality risk in wetter conditions. The high diarrheal mortality risk might also be related to extreme climate-related events like flooding, monsoons, and tropical cyclones, which both occur in tropical locations,^33^ because they could negatively impact water and sanitation infrastructure and overall immediate accessibility of health care that could potentially lead to serious diarrheal outcomes.^34–36^ An alternative explanation for our findings is that tropical regions do not get as dry over longer periods as arid and temperate regions inhibiting the concentration of pathogens in the environment. Conceptually, a U-shaped association between precipitation and diarrhea could occur in tropical climate as previously reported in studies from Bangladesh and Pacific Islands.^22,37^

In our findings, temperate and arid climates have U-shaped precipitation–diarrheal mortality associations depicting increased diarrheal mortality risk in both drier and wetter conditions. A study from a temperate location in China also demonstrated the U-shaped precipitation–diarrheal mortality associations.^38^ During dry conditions, less clean water is available for drinking and maintaining proper handwashing and disposal of feces that could lead to increased exposure to pathogens.^10^ Flow of water is also slow during extremely dry conditions that could increase the concentration of pathogens especially in locations near livestock.^39^ Additionally, piped water outages could be triggered by extremely dry conditions, leading to the usage of water with poor quality.^40^ The slightly higher diarrheal mortality risk in arid regions compared with temperate regions could be explained by its aridity, increasing the concentration of enteric pathogens in the environment and possibly rendering poorer sanitation and hand hygiene.^41,42^

The precipitation–diarrheal mortality associations were regionally different. In tropical climates, Asian countries, such as India, the Philippines, and Thailand, had high diarrheal mortality risk in extremely wet conditions unlike in Latin American countries or Brazil, Costa Rica, and Peru, wherein diarrheal mortality risks weakened toward extremely wet conditions. For arid and temperate climates, only South Africa had high diarrheal mortality risk in extremely wet conditions unlike the rest of the countries. Generally, these regional differences could be explained by regional water and sanitation conditions, socioeconomic inequality, and environmental landscapes that enable/prevent the transmission of diarrheal diseases.^16,43^ For tropical regions, the frequent occurrence of flooding, monsoons, and tropical cyclones in Asian countries could also contribute to the regional difference from Latin American countries.^44,45^

Our study utilized multiyear mortality data, determined by trained medical practitioners, at subnational units of several middle-income countries. Diarrheal mortality reflects severe diarrhea cases that could have different disease pathways or physical/environmental conditions from less severe diarrhea cases that do not lead to death. Thus, our use of mortality as outcome could primarily explain the discrepancies between our findings and those of Dimitrova et al. who used caregiver-reported cases. The survey periods of DHS data might have favored conditions or seasons conducive to data collection that could avoid capturing cases from very wet or dry conditions. Moreover, the diarrheal data of Dimitrova et al. are mostly from Sub-Saharan African locations categorized by third-level climate zone. Their use of finer spatial resolution and climate zone could have captured effects within smaller areas compared with ours that used larger spatial units and pooled estimates at first-level climate zone.

The exposure definition of choice is 28 days of cumulative precipitation before the diarrheal mortality, and it was intended to represent precipitation accumulation or deficiency in the environment. We hypothesize that intense wetness or dryness for about a month could indicate poorer environmental conditions, such as lower quality of drinking water and sanitation and less accessibility/availability of healthcare services, which enable multiple diarrheal infections and further worsen the health conditions of individuals already suffering from diarrheal diseases. Our exposure definition could not separately assess the events with heavy precipitation after long dry periods (e.g., 30–60 days), as demonstrated in other precipitation–diarrheal mortality studies.^28,46,47^ Such dry-to-heavy-precipitation events, which occur infrequently, could be hidden in our exposure definition as regular wet rather than extremely wet days. Although exploring this type of precipitation exposure is worthwhile, it may only explain a greater risk of diarrheal infections rather than more serious health outcomes like mortality, which may take place in severe conditions.

Our study has several limitations. First, considering that precipitation events are highly localized, the within-country climatic region aggregation we used can result in exposure measurement error. More exposure measurement error could be introduced for South Africa and Argentina as their data were aggregated using the first administrative level boundaries, which are relatively large geographical areas, even though the exposures and climate zone were determined based on population weights. The rest of the study sites were available at relatively small administrative levels, allowing finer spatial extraction of exposure and classification of climate zone. Second, the diarrheal mortality data lacked specific information on enteric pathogens, which could exhibit differing associations with precipitation. A recent study reported that several viral and bacterial pathogens (e.g., astrovirus, norovirus, sapovirus, and enterotoxigenic *E. coli*) favor drier conditions, defined by prior negative 7-day precipitation anomalies with lag of 3–9 days, and none showed increased risk under wetter conditions.^48^ Other studies indicated increased risk in wetter conditions and heavy precipitation for bacteria and protozoal pathogens like *Campylobacter*, *Vibrio cholerae*, *Cryptosporidium parvum*, and *Giardia lamblia*.^49–51^ Third, the years of diarrheal mortality data are different across countries with some starting before 2000 and some ending before 2019. This may have contributed to the remaining heterogeneity of the pooled estimates because diarrheal mortality risk has reduced over time due to better socioeconomic and environmental conditions. Fourth, the European Centre for Medium-Range Weather Forecasts climate re-analysis 5th generation land component precipitation data do not distinguish between rain and snow. Diarrhea risk may not follow the same transmission pathway after heavy rainfall and snowfall. Although there were no cold and polar regions included in our study, some temperate and arid study locations may have experienced snowfall and may have affected the shape of the precipitation–diarrheal mortality associations. Lastly, our results only included middle-income countries (i.e., 6 upper-middle-income and 2 lower-middle-income countries) and cannot represent conditions from high- and low-income countries that might have no/very low and very high diarrheal mortality, respectively.

In summary, our findings suggest that associations between precipitation and diarrheal mortality vary according to climate zones. We specifically found that diarrheal mortality risk increases in both drier and wetter conditions for temperate and arid climates, while increased diarrheal mortality risk only occurred in wetter conditions for tropical climates. These results could be used to develop global projections of future precipitation-related diarrheal mortality under various climate-related factors affecting diarrheal mortality, including ambient temperature.

## Conflict of interest statement

The authors declare that they have no conflicts of interest with regard to the content of this report.

## Acknowledgements

We thank the reviewers for their vital feedback and for helping improve the readability and clarity of this report.

## Supplementary Material


