# Supplementary material for: Association between precipitation and mortality due to diarrheal diseases by climate zone: A multi-country modeling study
Source: Environ Epidemiol. 2024 Jul 17;8(4):e320. doi: 10.1097/EE9.0000000000000320 (PMC11257672; doi:10.1097/EE9.0000000000000320)
Supplement: Supplementary file 1 [file ee9-8-e320-s001.pdf]

SUPPLEMENTAL DOCUMENT

Association between precipitation and mortality due to diarrheal diseases by climate zone: a multi-country modelling study

Table of Contents

Exposure data extraction (data aggregation).. 2

List of Tables

Table 1. Diarrheal mortality data information 5
Table 2. Data collected from open sources.. 6
Table 3. Köppen–Geiger climate classifications.. 8
Table 4. Summary of statistics by included and excluded within-country climatic regions........... 9
Table 5. List of R packages used for data analysis...12
Table 6. Minimum risk precipitation and percentage change of diarrhea mortality risk at dry (5th percentile precipitation) and wet (95th percentile precipitation) conditions by country and climate zone...13
Table 7. Sensitivity analysis for second stage modelling by incorporating relevant meta-predictors...14

List of Figures

Figure 1. Average annual rates of diarrheal deaths by sub-national units..................................15
Figure 2. Within-country climatic regions in 8 middle-income countries...................................16
Figure 3. Crude diarrheal deaths by within-country climatic region...18
Figure 4. Monthly average diarrheal mortality and 28-day cumulative precipitation by included within-country climatic regions 19
Figure 5. Sensitivity analysis of precipitation–diarrheal mortality associations by changing 3-month time stratification 20
Figure 6. Sensitivity analysis of precipitation–diarrheal mortality associations by changing to 1-month time stratification 21
Figure 7. Sensitivity analysis of precipitation–diarrheal mortality associations by reducing number of precipitation running days 22
Figure 8. Sensitivity analysis of precipitation–diarrheal mortality associations by removing month indicator 23
Figure 9. Sensitivity analysis of precipitation–diarrheal mortality associations by incorporating various meta-predictors...24
Figure 10. Sensitivity analysis of precipitation–diarrheal mortality associations by changing the percentage of improved sanitation coverage 26
Figure 11. Sensitivity analysis of precipitation–diarrheal mortality associations by changing the percentage of piped water coverage 27

Exposure data extraction (data aggregation)
We explain here in detail the steps in extracting the population-weighted precipitation and temperature from ERA5-Land. R codes used are in GitHub repository of the corresponding author (https://github.com/paulcarlos).

1. Data download from Copernicus Website:
   1.1. NetCDF-3 files of ERA5-Land 2-meter temperatures and total precipitation,[1] in 0.1 degree (~9km) grids from the Copernicus Climate Change Service website (https://cds.climate.copernicus.eu/cdsapp#!/dataset/reanalysis-era5-land?tab=form) for each country using the following extent coordinates and time periods:

Country	North (maximum latitude)	West (minimum longitude)	South (minimum latitude)	East (maximum longitude)	Years
Argentina	-21	-74	-56	-53	2004–2016
Brazil	6	-75	-34	-34	1999–2020
Costa Rica	12	-88	5	-82	2013–2020
India	38	65	5	99	2000–2014
Peru	0	-82	-19	-68	2002–2020
Philippines	23	113	4	128	2005–2020
South Africa	33	-35	16	-21	1996–2014
Thailand	21	97	5	106	2010–2020

   1.2. NetCDF file of Gridded Population of the World version 4.11 for UN WPP-adjusted population density in 2.5 arc minute (~5km) grids from the Centre for International Earth Science Information Network website (https://sedac.ciesin.columbia.edu/data/set/gpw-v4-population-density-adjusted-to-2015-unwpp-country-totals-rev11/data-download).
   1.3. TIF file of global raster of present Koppen-Geiger climate classification for years 1980–2016 in 0.083 degree (~9km) grids from GloH20 website (https://www.gloh2o.org/koppen/).[2]
   1.4. Administrative boundaries of locations for each country:

Country	Administrative level	Format	Source
Argentina	Province	Polygons	Database of Global Administrative Areas (GADM) version 4.1
Brazil	Municipality	Polygons	Instituto Brasileiro de Geografia e Estatística version 2017
Costa Rica	Province	Polygons	Database of Global Administrative Areas (GADM) version 4.1
India	Village for rural areas and Postal Index Number codes for urban areas	Coordinates	Centre for Global Health Research
Peru	Province	Polygons	Database of Global Administrative Areas (GADM) version 4.1

Philippines	City or municipality	Polygons	Humanitarian Data Exchange (HDX)
South Africa	District	Polygons	Database of Global Administrative Areas (GADM) version 4.1
Thailand	District	Polygons	Database of Global Administrative Areas (GADM) version 4.1

2. Population and climate classification rasters were re-sampled to align with ERA5-Land rasters:
   2.1. Average population density rasters across available years to form a single raster per country:
      2.1.1. Argentina: 2005, 2010, 2015
      2.1.2. Brazil: 2000, 2005, 2010, 2015, 2020
      2.1.3. Costa Rica: 2015, 2020
      2.1.4. India: 2000, 2005, 2010, 2015
      2.1.5. Peru: 2000, 2005, 2010, 2015, 2020
      2.1.6. Philippines: 2005, 2010, 2015, 2020
      2.1.7. South Africa: 2000, 2005, 2010, 2015
      2.1.8. Thailand: 2010, 2015, 2020
   2.2. Aligned (re-sampling) population density and climate rasters using the bilinear and nearest neighbour methods, respectively, following to the ERA5-Land raster in ~9km grids for each country.

3. Matrix/raster for each administrative boundary/sub-national unit were created following ERA5-Land raster/grids. The following were the steps performed:
   3.1. Imported a sample (1 hour) raster of ERA5-Land data (either temperature or precipitation) and polygons of administrative boundaries for each country
   3.2. Created blank matrix with zero (0) values following the dimensions (longitude and latitude values) of ERA5-Land raster per administrative boundary/sub-national unit
   3.3. Selected cells or grids of ERA5-Land raster that intersects with polygon of administrative boundary and assign "1" value. For large administrative area, grid centroids within the polygons were selected. For small administrative areas, any intersecting grids with the polygons were selected.
   3.4. Outputs are matrices of each administrative boundary of locations per country following the format/dimensions of ERA5-Land raster
   3.5. Performed "for loops" per administrative boundary of a country

4. Each administrative boundary or sub-national unit (e.g., province, district, municipality) was given a sub-climate classification (population weighted) using the following steps:
   4.1. 30 sub-climate classifications,[3] were used to assign each administrative boundary
   4.2. Imported re-sampled rasters of sub-climate classification, population, and administrative boundaries/sub-national units per country
   4.3. Selected cells or grids of sub-climate classification and population rasters that are part of the administrative boundary
   4.4. Calculated the sum of population for each sub-climate classification among the set of grids of the administrative boundary/sub-national unit
   4.5. Used the sub-climate classification of the highest population to represent an administrative boundary

5. Daily 2-meter temperatures and total precipitation for each climate zone of a country were derived using the following steps:
   5.1. Extracted hourly (UTC +0:00) population-weighted averages of 2-meter temperatures and total precipitation for each administrative boundary based on the ERA5-Land, population, and administrative boundary rasters. Population density served as weights in taking averages per administrative boundary.
   5.2. Aggregated hourly values (mean for temperatures and sum for precipitation) into daily values by grid square following local time zones:

**Country**	**Time zones**
Argentina	"America/Buenos_Aires"
Brazil	"America/Belem", "America/Manaus", "America/Rio_Branco"
Costa Rica	"America/Costa Rica"
India	"Asia/Kolkata"
Peru	"America/Lima"
Philippines	"Asia/Manila"
South Africa	"Africa/Johannesburg"
Thailand	"Asia/Bangkok"

   5.3. Aggregated daily values by within-country climatic regions by calculating mean of daily precipitation or temperatures across sub-national units with the same sub-climate zone and using diarrheal deaths as weights.

Table 1. Diarrheal mortality data information

Country	Data source	ICD10 codes[a]	Total no. of deaths	Years included	Location of death[b]
Argentina	Instituto Nacional de Estadística y Censos	A01, A02, A03, A04, A05, A06, A08, A09	3,886	2005–2015	24 provinces
Brazil	Instituto Brasileiro de Geografia e Estatística	A00, A01, A02, A03, A04, A05, A06, A07, A08, A09	101,872	2000–2021	3,850 municipalities
Costa Rica	Instituto Nacional de Estadística y Censos	A00, A02, A03, A04, A05, A06, A07, A08, A09	859	2014–2021	7 provinces
India	Million Death Study[4]	A09	30,644	2001–2013	7,868 locations[c]
Peru	Instituto Nacional de Estadística e Informática	A00, A01, A02, A03, A04, A05, A06, A07, A08, A09	8,057	2003–2020	190 provinces
Philippines	Philippine Statistics Authority	A00, A01, A02, A03, A04, A05, A06, A07, A08, A09	65,978	2006–2019	1,530 municipalities/ cities
Thailand	National Statistical Office	A00, A01, A02, A03, A04, A05, A06, A07, A08, A09	17,852	2011–2020	890 districts
South Africa	Statistics South Africa	A00, A01, A02, A03, A04, A05, A06, A07, A08, A09	380,355	1997–2013	52 districts

[a]10th revision of the International Classification of Diseases
[b]Smallest administrative level available
[c]Village for rural areas and PIN codes for urban areas

Table 2. Data collected from open sources

Variable	Description	Data source
Precipitation	Historical gridded hourly total precipitation in meters and UTC +00:00 from 1996 to 2021	ERA5-Land dataset was downloaded from the ECMWF Climate Data Store website (https://cds.climate.copernicus.eu/). ERA5-Land is a replay of the land component of the ERA5 climate reanalysis at an enhanced resolution of ~9 km that is evaluated with in situ observations from 2001–2018 and additional models or satellite-based global reference datasets.[1]
Temperature	Historical gridded hourly 2-meter air temperatures in Kelvin and UTC +00:00 from 1996 to 2021	
Climate classification	Gridded Köppen-Geiger climate classifications for present day based on 1980-2016 data and includes 30 classifications (shown in Table 3)	The updated and downscaled Köppen-Geiger climate classification raster maps were produced by Beck et al. (2018).[2] The data was downloaded from the GloH20 website (https://www.gloh2o.org/koppen/). The raster maps are available in 0.5°, 0.083°, and 0.0083° resolution. The raster map with 0.083° or ~9km grids was selected.
Population	Historical gridded population density (population per km^2) in 2000, 2005, 2010, 2015, and 2020	Gridded Population of the World version 4.11 dataset was downloaded from the Centre for International Earth Science Information Network website (https://sedac.ciesin.columbia.edu/data/set/gpw-v4-population-count-rev11). The gridded population adjusted for 2015 United Nations' World Population Prospects values were used. The data was available in ~5 km grid resolution.
Gross domestic product (GDP)	Gridded GDP per capita for purchasing power parity expressed in 2011 international US$ from 1990 to 2015	Gridded global dataset for Gross Domestic Product was produced by Kummu et al. (2018).[5] The data was downloaded from https://doi.org/10.5061/dryad.dk1j0.
Access to improved sanitation	Percentage of household with access to improved sanitation in low-middle income countries from 2000 to 2017	Downscaled water and sanitation indicators for selected low-middle income countries were produced by the Institute of Health Metrics and Evaluation (IHME).[6] The data was downloaded from IHME's website (https://cloud.ihme.washington.edu/s/bkH2X2tFQMejMxy). The data was available in ~5km grid resolution. Argentina was not included in the IHME's data, so the same water and sanitation indicators were downloaded from the UNICEF's country level dataset (https://data.unicef.org/).
Open defection	Percentage of household with practising open defecation in low-middle income countries from 2000 to 2017	
Access to improved drinking water sources	Percentage of household with access to improved drinking water sources in low-middle income countries from 2000 to 2017	
Piped water	Percentage of household with piped water connection in low-	

	middle income countries from 2000 to 2017	

Note: All the gridded datasets were bilinearly re-sampled except for Köppen-Geiger climate classification in which nearest neighbour re-sampling was used to align with the ~9 km grids of the ERA5-Land data. We used projectRaster function from raster R package to re-sample rasters.

Table 3. Köppen–Geiger climate classifications

No.	Main climate (1st-level classification)	Sub-climate (3rd-level classification)	Symbols
1	Tropical	Rainforest	Af
2	Tropical	Monsoon	Am
3	Tropical	Savannah	Aw
4	Arid	Desert, Hot	BWh
5	Arid	Desert, Cold	BWk
6	Arid	Steppe, Hot	BSh
7	Arid	Steppe, Cold	BSk
8	Temperate	Dry Summer, Hot Summer	Csa
9	Temperate	Dry Summer, Warm Summer	Csb
10	Temperate	Dry Summer, Cold Summer	Csc
11	Temperate	Dry Winter, Hot Summer	Cwa
12	Temperate	Dry Winter, Warm Summer	Cwb
13	Temperate	Dry Winter, Cold Summer	Cwc
14	Temperate	No Dry Season, Hot Summer	Cfa
15	Temperate	No Dry Season, Warm Summer	Cfb
16	Temperate	No Dry Season, Cold Summer	Cfc
17	Cold	Dry Summer, Hot Summer	Dsa
18	Cold	Dry Summer, Warm Summer	Dsb
19	Cold	Dry Summer, Cold Summer	Dsc
20	Cold	Dry Summer, Very Cold Winter	Dsd
21	Cold	Dry Winter, Hot Summer	Dwa
22	Cold	Dry Winter, Warm Summer	Dwb
23	Cold	Dry Winter, Cold Summer	Dwc
24	Cold	Dry Winter, Very Cold Winter	Dwd
25	Cold	No Dry Season, Hot Summer	Dfa
26	Cold	No Dry Season, Warm Summer	Dfb
27	Cold	No Dry Season, Cold Summer	Dfc
28	Cold	No Dry Season, Very Cold Winter	Dfd
29	Polar	Tundra	ET
30	Polar	Frost	EF

Based on Peel et al.[3]

Table 4. Summary of statistics by included and excluded within-country climatic regions

No.	Inclusion	ID	Country	Main climate	Sub-climate	Year	Total deaths	Average daily deaths	Range of daily deaths	Average daily 28-day precipitation sums	Range of daily 28-day precipitation sums	Average daily temperatures
1.	excluded	arg.Aw	Argentina	Tropical	Tropical, savannah	2005–2015	232	0.1	0–2	1398	39–5758	22.9
2.	excluded	arg.BSh	Argentina	Arid	Arid, steppe, hot	2005–2015	194	0	0–2	878	10–3596	20.1
3.	excluded	arg.BSk	Argentina	Arid	Arid, steppe, cold	2005–2015	71	0	0–1	606	5–2816	17.4
4.	excluded	arg.BWk	Argentina	Arid	Arid, desert, cold	2005–2015	565	0.1	0–2	511	77–1820	13.2
5.	included	arg.Cfa	Argentina	Temperate	Temperate, no dry season, hot summer	2005–2015	1729	0.4	0–17	1193	118–3971	19.7
6.	included	arg.Cwa	Argentina	Temperate	Temperate, dry winter, hot summer	2005–2015	1083	0.3	0–4	1258	27–4208	16.5
7.	excluded	arg.ET	Argentina	Polar	Polar, tundra	2005–2015	8	0	0–1	648	186–1703	4
8.	included	bra.Af	Brazil	Tropical	Tropical, rainforest	2000–2019	7624	1	0–8	1637	504–3729	24.9
9.	included	bra.Am	Brazil	Tropical	Tropical, monsoon	2000–2019	13768	1.9	0–10	1329	367–4014	25.4
10.	included	bra.Aw	Brazil	Tropical	Tropical, savannah	2000–2019	40283	5.5	0–21	947	126–3696	24.7
11.	included	bra.BSh	Brazil	Arid	Arid, steppe, hot	2000–2019	7654	1	0–10	457	36–4321	25.3
12.	included	bra.Cfa	Brazil	Temperate	Temperate, no dry season, hot summer	2000–2019	17147	2.3	0–10	1299	139–3211	19.9
13.	included	bra.Cfb	Brazil	Temperate	Temperate, no dry season, warm summer	2000–2019	3751	0.5	0–5	1315	102–3049	17.9
14.	included	bra.Cwa	Brazil	Temperate	Temperate, dry winter, hot summer	2000–2019	3598	0.5	0–4	1093	3–4658	20.4
15.	excluded	bra.Cwb	Brazil	Temperate	Temperate, dry winter, warm summer	2000–2019	331	0	0–2	1175	2–4770	19.1
16.	excluded	cri.Af	Costa Rica	Tropical	Tropical, rainforest	2014–2019	51	0	0–1	2687	235–9200	23.1
17.	included	cri.Am	Costa Rica	Tropical	Tropical, monsoon	2014–2019	610	0.3	0–4	2224	134–7724	20.6
18.	excluded	cri.Aw	Costa Rica	Tropical	Tropical, savannah	2014–2019	67	0	0–2	1288	62–5541	25.7
19.	included	ind.Am	India	Tropical	Tropical, monsoon	2001–2013	1583	0.3	0–6	2665	6–11100	25.1
20.	included	ind.Aw	India	Tropical	Tropical, savannah	2001–2013	9794	2.1	0–31	1265	8–4763	26.2
21.	included	ind.BSh	India	Arid	Arid, steppe, hot	2001–2013	5981	1.3	0–14	714	0–3182	25.7
22.	excluded	ind.BSk	India	Arid	Arid, steppe, cold	2001–2013	6	0	0–1	843	20–3053	-6
23.	excluded	ind.BWh	India	Arid	Arid, desert, hot	2001–2013	423	0.1	0–3	344	0–3016	26.1
24.	excluded	ind.Cfa	India	Temperate	Temperate, no dry season, hot summer	2001–2013	47	0	0–2	1600	34–6622	10.4
25.	excluded	ind.Csa	India	Temperate	Temperate, dry summer, hot summer	2001–2013	30	0	0–1	1402	29–5993	11.1
26.	included	ind.Cwa	India	Temperate	Temperate, dry winter, hot summer	2001–2013	12445	2.6	0–24	1439	5–5688	23.6

No.	Inclusion	ID	Country	Main climate	Sub-climate	Year	Total deaths	Average daily deaths	Range of daily deaths	Average daily 28-day precipitation sums	Range of daily 28-day precipitation sums	Average daily temperatures
27.	excluded	ind.Cwb	India	Temperate	Temperate, dry winter, warm summer	2001–2013	243	0.1	0–2	3321	291–10216	13.4
28.	excluded	ind.Dfb	India	Cold	Cold, no dry season, warm summer	2001–2013	47	0	0–2	1609	39–5666	6
29.	excluded	ind.Dsb	India	Cold	Cold, dry summer, warm summer	2001–2013	11	0	0–1	1174	22–4562	1.8
30.	excluded	ind.Dwb	India	Cold	Cold, dry winter, warm summer	2001–2013	10	0	0–1	1652	39–5589	3.9
31.	excluded	ind.ET	India	Polar	Polar, tundra	2001–2013	11	0	0–1	932	39–2834	-5.6
32.	included	per.Af	Peru	Tropical	Tropical, rainforest	2003–2019	618	0.1	0–3	2138	381–4794	21.7
33.	excluded	per.Am	Peru	Tropical	Tropical, monsoon	2003–2019	217	0	0–2	2168	184–5499	23.7
34.	excluded	per.Aw	Peru	Tropical	Tropical, savannah	2003–2019	210	0	0–2	1641	396–4901	17.5
35.	excluded	per.BSk	Peru	Arid	Arid, steppe, cold	2003–2019	46	0	0–1	990	9–3719	13
36.	included	per.BWh	Peru	Arid	Arid, desert, hot	2003–2019	2910	0.5	0–5	274	38–3046	20.3
37.	excluded	per.BWk	Peru	Arid	Arid, desert, cold	2003–2019	557	0.1	0–3	428	31–3156	14
38.	excluded	per.Cfb	Peru	Temperate	Temperate, no dry season, warm summer	2003–2019	298	0	0–2	1312	276–5568	14.1
39.	excluded	per.Cwb	Peru	Temperate	Temperate, dry winter, warm summer	2003–2019	538	0.1	0–3	1234	71–3302	10.7
40.	excluded	per.ET	Peru	Polar	Polar, tundra	2003–2019	2155	0.3	0–5	1266	74–3425	7.6
41.	included	phl.Af	Philippines	Tropical	Tropical, rainforest	2006–2019	27665	5.4	0–18	3053	350–6528	25.8
42.	included	phl.Am	Philippines	Tropical	Tropical, monsoon	2006–2019	19984	3.9	0–14	2750	168–7014	26
43.	included	phl.Aw	Philippines	Tropical	Tropical, savannah	2006–2019	17490	3.4	0–16	2537	91–8685	26.3
44.	excluded	phl.Cwb	Philippines	Temperate	Temperate, dry winter, warm summer	2006–2019	147	0	0–2	3842	126–11483	18.6
45.	excluded	tha.Af	Thailand	Tropical	Tropical, rainforest	2011–2019	126	0	0–2	2811	126–8424	25.3
46.	included	tha.Am	Thailand	Tropical	Tropical, monsoon	2011–2019	1482	0.5	0–4	2386	96–6579	26.2
47.	included	tha.Aw	Thailand	Tropical	Tropical, savannah	2011–2019	14018	4.3	0–16	1455	6–4665	26.5
48.	excluded	tha.Cwa	Thailand	Temperate	Temperate, dry winter, hot summer	2011–2019	4	0	0–1	1692	0–6205	21.2
49.	included	zaf.BSh	South Africa	Arid	Arid, steppe, hot	1997–2013	31453	5.1	0–24	561	16–2493	18.7
50.	included	zaf.BSk	South Africa	Arid	Arid, steppe, cold	1997–2013	48636	7.8	0–34	500	1–2329	18.3
51.	excluded	zaf.BWk	South Africa	Arid	Arid, desert, cold	1997–2013	160	0	0–2	294	7–1738	16.9
52.	included	zaf.Cfa	South Africa	Temperate	Temperate, no dry season, hot summer	1997–2013	75071	12.1	0–46	843	27–4133	18.6
53.	included	zaf.Cfb	South Africa	Temperate	Temperate, no dry season, warm summer	1997–2013	15548	2.5	0–15	844	12–2986	16.1

No.	Inclusion	ID	Country	Main climate	Sub-climate	Year	Total deaths	Average daily deaths	Range of daily deaths	Average daily 28-day precipitation sums	Range of daily 28-day precipitation sums	Average daily temperatures
54.	included	zaf.Csa	South Africa	Temperate	Temperate, dry summer, hot summer	1997–2013	1988	0.3	0–7	536	24–2357	16.6
55.	included	zaf.Csb	South Africa	Temperate	Temperate, dry summer, warm summer	1997–2013	5607	0.9	0–7	619	27–3057	16.5
56.	included	zaf.Cwa	South Africa	Temperate	Temperate, dry winter, hot summer	1997–2013	106263	17.1	0–53	702	9–3548	18.7
57.	included	zaf.Cwb	South Africa	Temperate	Temperate, dry winter, warm summer	1997–2013	95629	15.4	0–54	749	3–3346	15.5

Note:

- Highlighted in yellow are included locations.
- Daily total precipitation and 28-day running sums of prior total precipitation are expressed in millimeters (mm).
- Daily mean temperatures are expressed in degree Celsius (°C).

Table 5. List of R packages used for data analysis

R package	Version	Purpose/application
ecmwfr	1.5.0	Downloading ERA5-Land climate variables
ncdf4	1.21	Manipulating NetCDF files of various gridded data
raster	3.6-23	Manipulating, extraction, and re-sampling of gridded data
sf	1.0-14	Manipulating polygon area boundaries and point coordinates
lubridate	1.9.2	Conversion of hours to dates and time zones
RcppRoll	0.3.0	Computing running sums and means
splines	4.3.1	Applying natural cubic B-splines
gnm	1.1-4	Applying time-stratified case crossover
dlnm	2.4.7	Applying cross-basis matrix
mixmeta	1.2.0	Applying multi-level meta-regression
ggplot2	3.4.3	Creating figures
cowplot	1.1.1	Creating figures
sjPlot	2.8.15	Creating figures
RColorBrewer	1.1-3	Selecting colors for figures
classInt	0.4-10	Applying jenks for the figures

Table 6. Minimum risk precipitation and percentage change of diarrhea mortality risk at dry (5th percentile precipitation) and wet (95th percentile precipitation) conditions by country and climate zone

No.	Country	Climate	Minimum risk precipitation		Highest risk precipitation				5th percentile precipitation			95th percentile precipitation		
			mm	pctl	mm	pctl	% change	95%CI	mm	% change	95%CI	mm	% change	95%CI
1	Argentina	Temperate	1139	49	108	0	4.4	0.9; 7.9	311	3.1	0.5; 5.7	2457	1.1	-6.9; 9.8
2	Brazil	Tropical	349	0	1828	86	13.4	7.1; 20.2	-	-	-	2200	12.1	4.2; 20.5
3	Brazil	Arid	632	76	37	0	10.1	4.1; 16.6	84	8.6	3.4; 14.1	1247	2.6	0.3; 4.9
4	Brazil	Temperate	1167	49	121	0	5.9	3.0; 9.0	342	4.0	1.9; 6.2	2246	-1.7	-5.9; 2.8
5	Costa Rica	Tropical	134	0	4609	91	15.4	5.8; 25.8	-	-	-	5261	15.2	2.4; 29.5
6	India	Tropical	9	0	7527	100	23.7	-0.8; 54.4	-	-	-	5569	19.3	7.4; 32.5
7	India	Arid	732	66	2281	93	5.4	-3.9; 15.6	29	4.2	0.7; 7.9	2496	5.2	-6.6; 18.6
8	India	Temperate	1476	65	6	0	3.1	0.9; 5.3	75	2.9	0.8; 4.9	4397	0.4	-9.2; 11.2
9	Peru	Tropical	382	0	3552	93	15.9	4.7; 28.3	-	-	-	3676	15.9	3.1; 30.1
10	Peru	Arid	410	81	39	0	8.0	0.7; 15.8	68	6.7	0.4; 13.3	979	3.6	0.5; 6.8
11	Philippines	Tropical	232	0	6490	100	39.1	14.2; 69.4	-	-	-	5031	24.0	14.1; 34.7
12	Thailand	Tropical	66	0	4134	100	34.7	6.8; 70.1	-	-	-	3344	22.9	11.6; 35.3
13	South Africa	Arid	517	56	2401	100	11.5	-7.5; 34.3	43	4.3	0.3; 8.4	1348	6.6	1.9; 11.5
14	South Africa	Temperate	657	47	1855	100	4.4	-3.5; 13	286	2.0	0.1; 3.9	1245	3.1	1.0; 5.3

mm=millimeters; pctl=percentile; % change = percentage change relative to minimum risk precipitation; CI=confidence interval

Table 7. Sensitivity analysis for second stage modelling by incorporating relevant meta-predictors

Model #	Fixed effects	Random effects	I^2 (%)	Cochran *Q* statistic (p-value)	Wald test (p-value) for climate	Wald test (p-value) with other predictor
1	Climate	Country	38.6	0.0004[a]	1.39×10^{-7} [a]	-
2	Climate + population density	Country	40.3	0.0002[a]	7.92×10^{-7} [a]	0.7832
3	Climate + GDP per capita	Country	37.1	0.0009[a]	3.04×10^{-5} [a]	0.1763
4	Climate + improved sanitation	Country	34.5	0.0022[a]	7.10×10^{-6} [a]	0.0100[a]
5	Climate + open defecation	Country	39.3	0.0004[a]	1.09×10^{-6} [a]	0.3985
6	Climate + improved water	Country	38.9	0.0004[a]	4.65×10^{-8} [a]	0.4049
7	Climate + piped water	Country	31.0	0.0067[a]	0.0335[a]	0.0095[a]

[a]Significant *p*-value <0.05

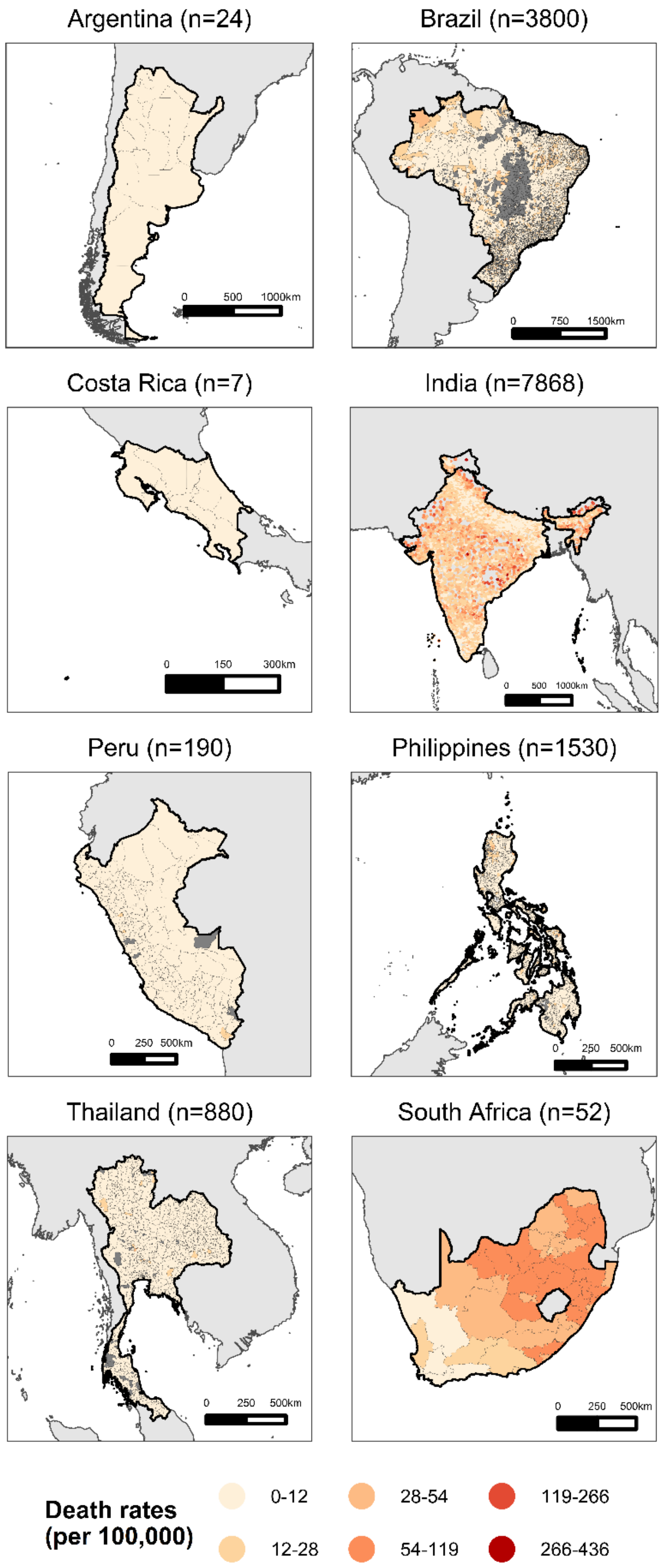

Figure 1. Average annual rates of diarrheal deaths by sub-national units
"n" refers to the number of sub-national units. Small Indian areas are depicted in coordinates. Color breaks generated using Jenks natural breaks.

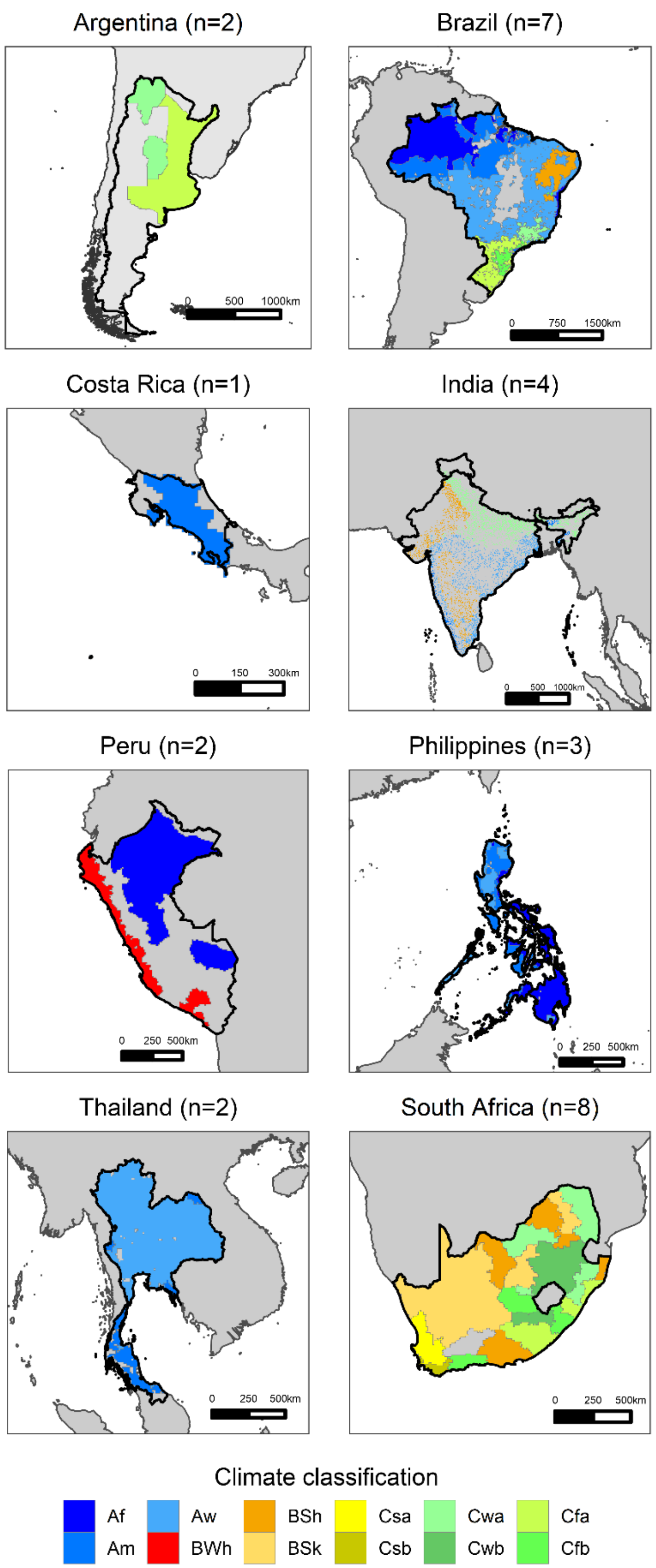

Figure 2. Within-country climatic regions in 8 middle-income countries
Köppen–Geiger climate classifications are: Af = Tropical Rainforest; Am = Tropical Monsoon; Aw = Tropical Savannah; BWh = Arid Desert, Hot; BSh = Arid Steppe, Hot; BSk = Arid Steppe, Cold;

Csa = Temperate Dry Summer, Hot Summer; Csb = Temperate Dry Summer, Warm Summer; Cwa = Temperate Dry Winter, Hot Summer; Cwb = Temperate Dry Winter, Warm Summer; Cfa = Temperate No Dry Season, Hot Summer; Cfb = Temperate No Dry Season, Warm Summer. “n” refers to number of included within-country climatic regions.

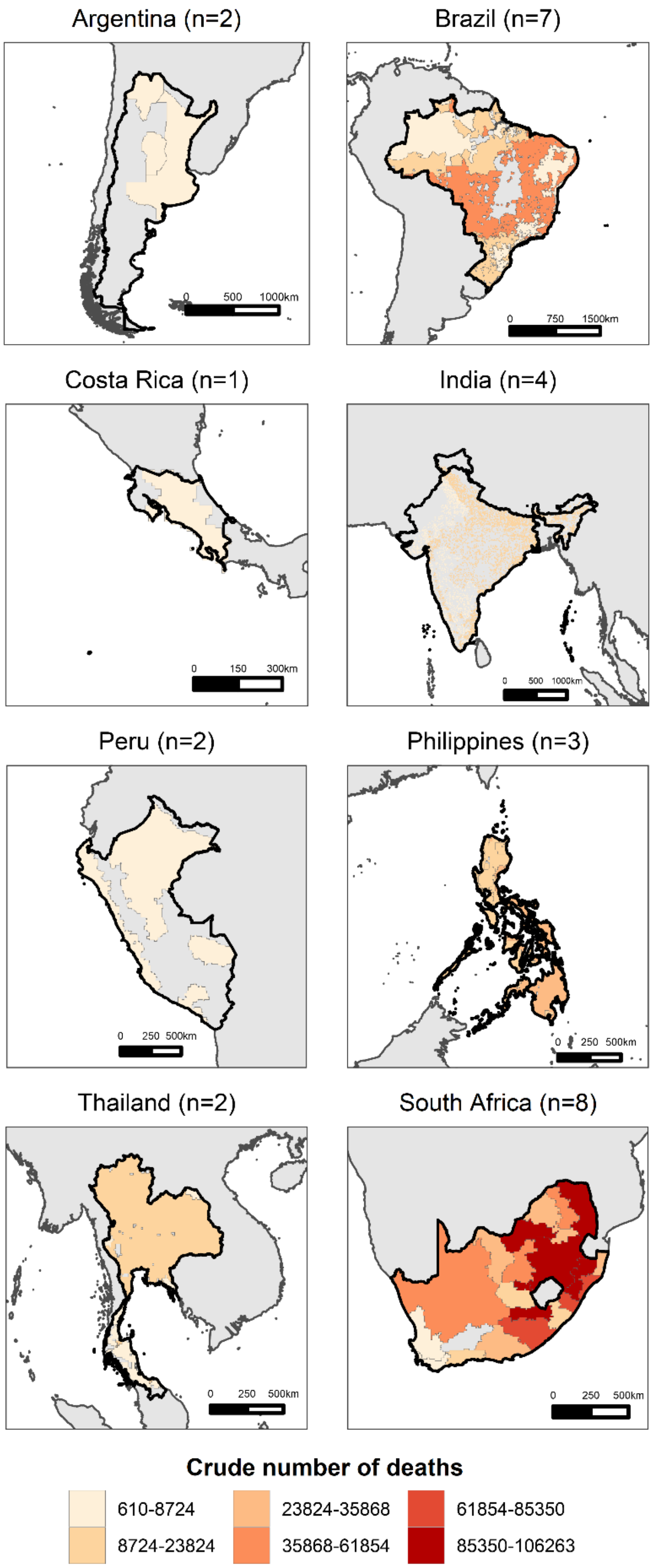

Figure 3. Crude diarrheal deaths by within-country climatic region
"n" refers to number of included within-country climatic regions. Color breaks generated using Jenks natural breaks.

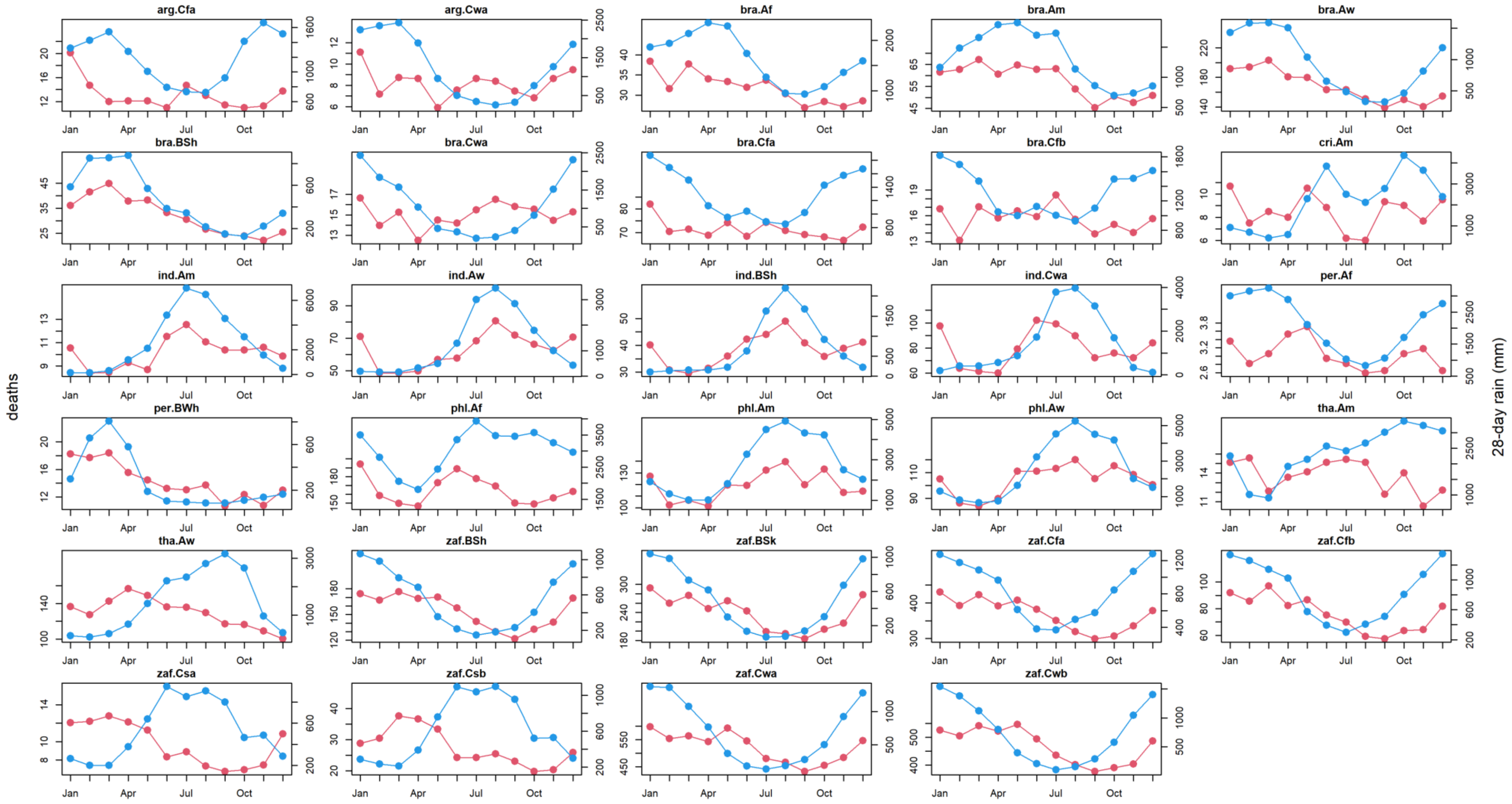

Figure 4. Monthly average diarrheal mortality and 28-day cumulative precipitation by included within-country climatic regions

Note:

- Primary Y-axis is average monthly diarrheal deaths, secondary Y-axis is average monthly 28-day running sums of total precipitation in mm, and X-axis is months.
- Red circles are diarrheal deaths and blue circles are precipitation values.
- Main titles of plots are a combination of 3-letter abbreviations of country names and sub-climate classifications shown in eTable 3.

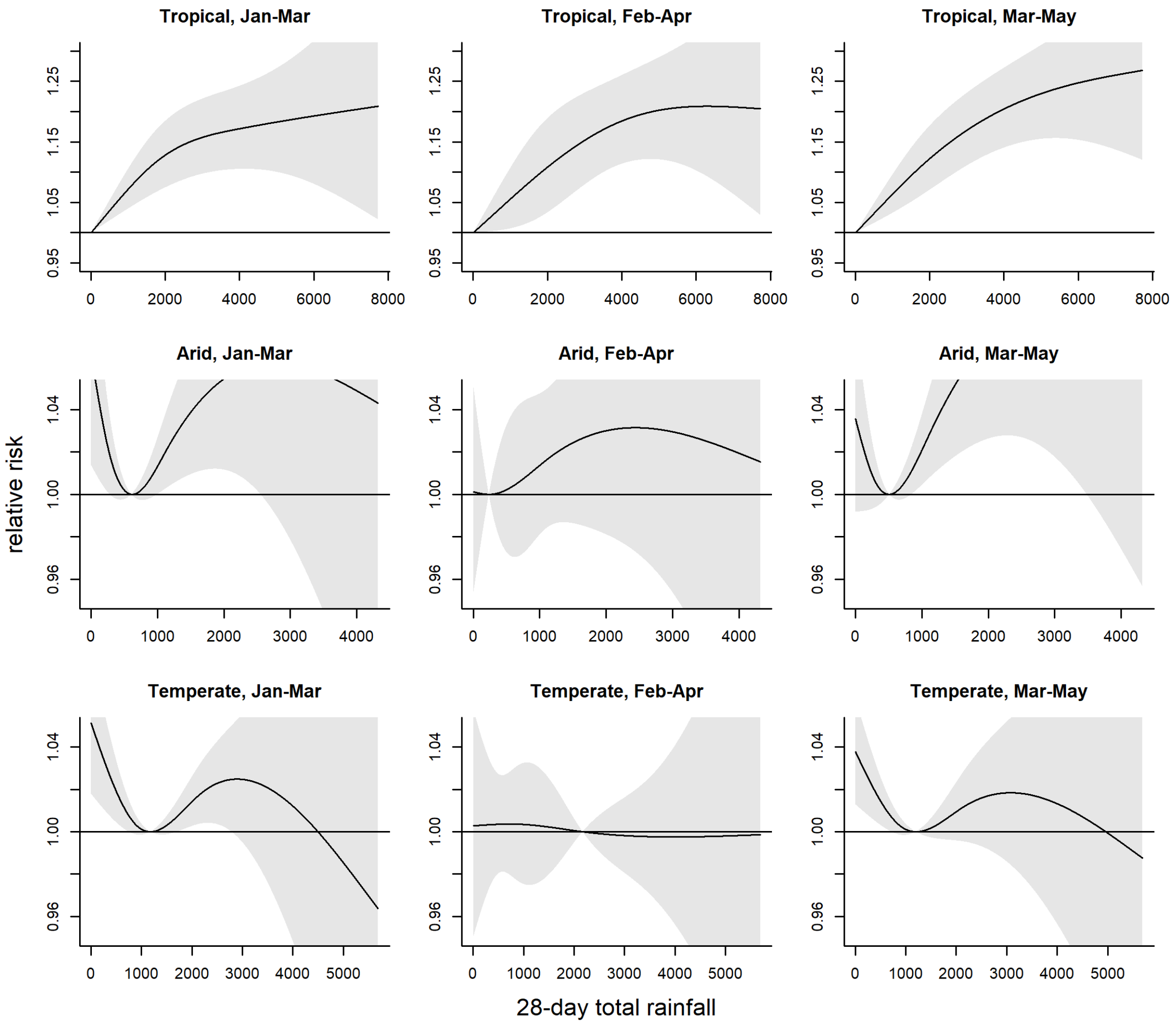

Figure 5. Sensitivity analysis of precipitation–diarrheal mortality associations by changing 3-month time stratification

“Jan-Mar” refers to 3-month stratification of January-March, April-June, July-September, October-December. “Feb-Apr” refers to 3-month stratification of February-April, May-July, August-October, November-January. “Mar-May” refers to 3-month stratification of March-May, June-August, September-November, December-February. Black solid horizontal line is relative risk 1.00.

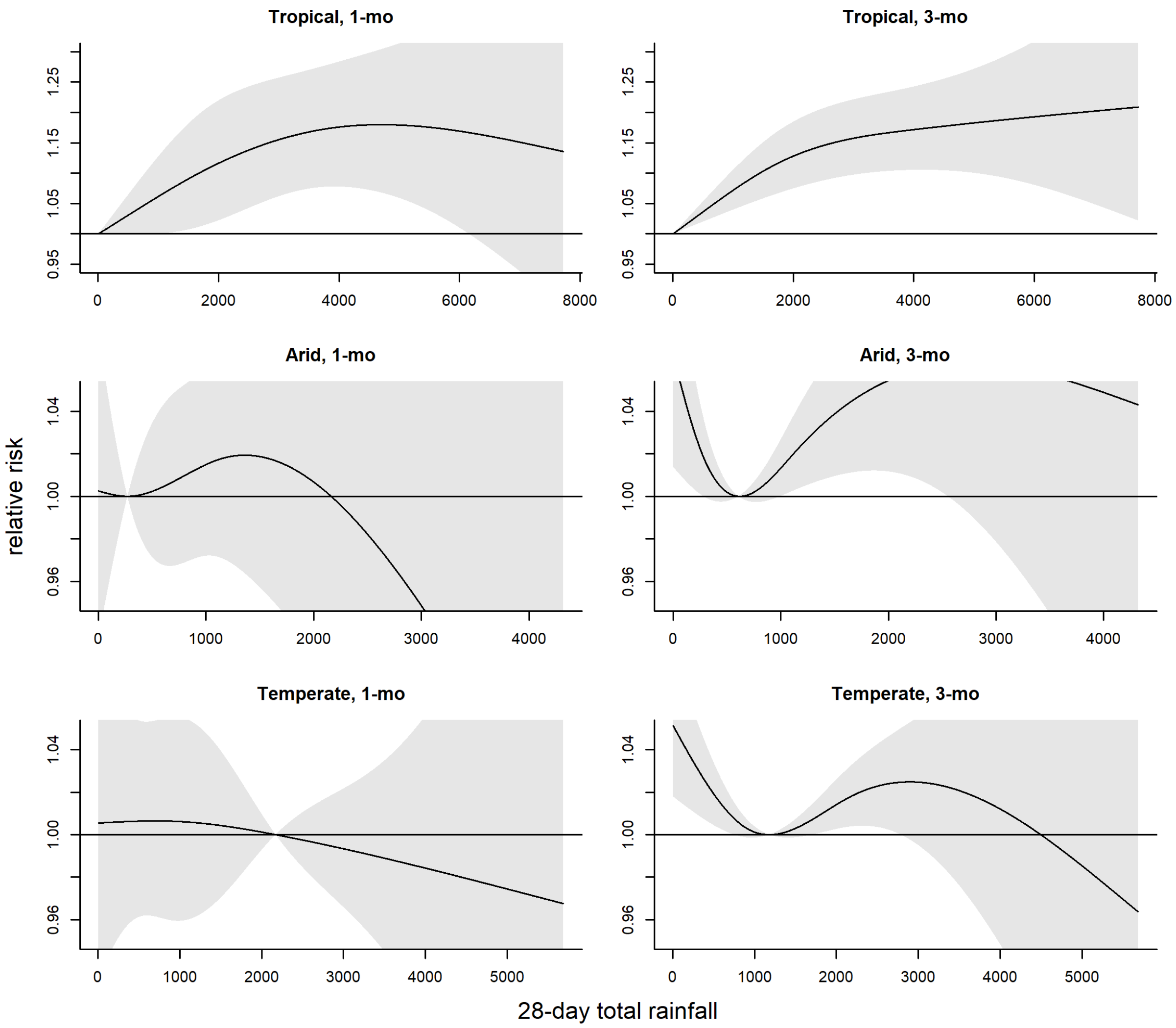

Figure 6. Sensitivity analysis of precipitation–diarrheal mortality associations by changing to 1-month time stratification
"1-mo" refers to time stratification of same year, month, and day-of-the-week. "3-mo" refers to the original time stratification of same year, 3-month, and day-of-the-week. Black solid horizontal line is relative risk 1.00.

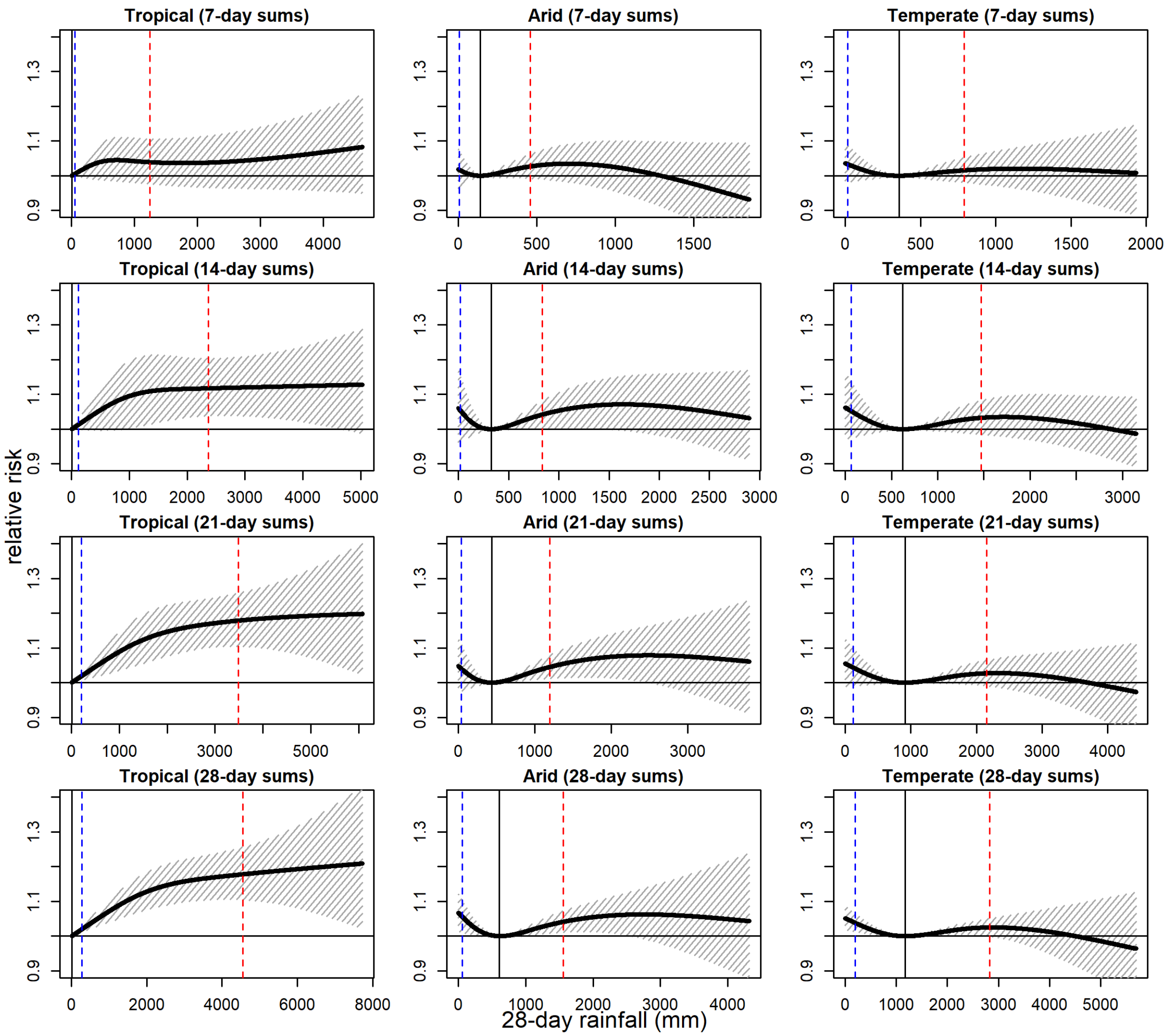

Figure 7. Sensitivity analysis of precipitation–diarrheal mortality associations by reducing number of precipitation running days

Black vertical lines are minimum risk precipitation. Blue and red broken lines are 5th percentile and 95th percentile of prior precipitation running sums. Black solid horizontal line is relative risk 1.00.

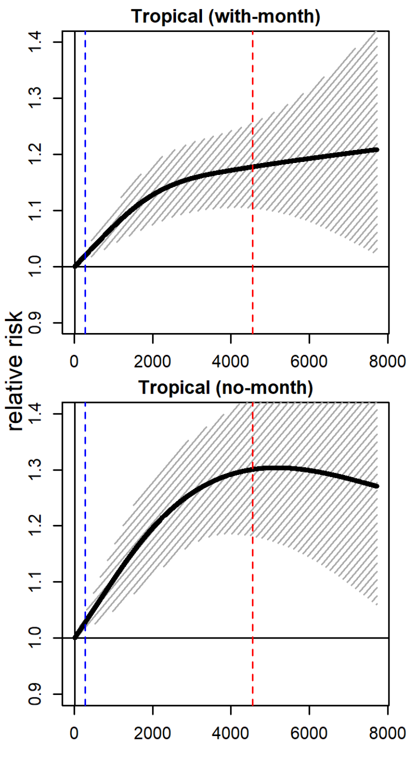

Figure 8. Sensitivity analysis of precipitation–diarrheal mortality associations by removing month indicator

Black vertical lines are minimum risk precipitation. Blue and red broken lines are 5th percentile and 95th percentile of prior 28-day precipitation running sums. Black solid horizontal line is relative risk 1.00.

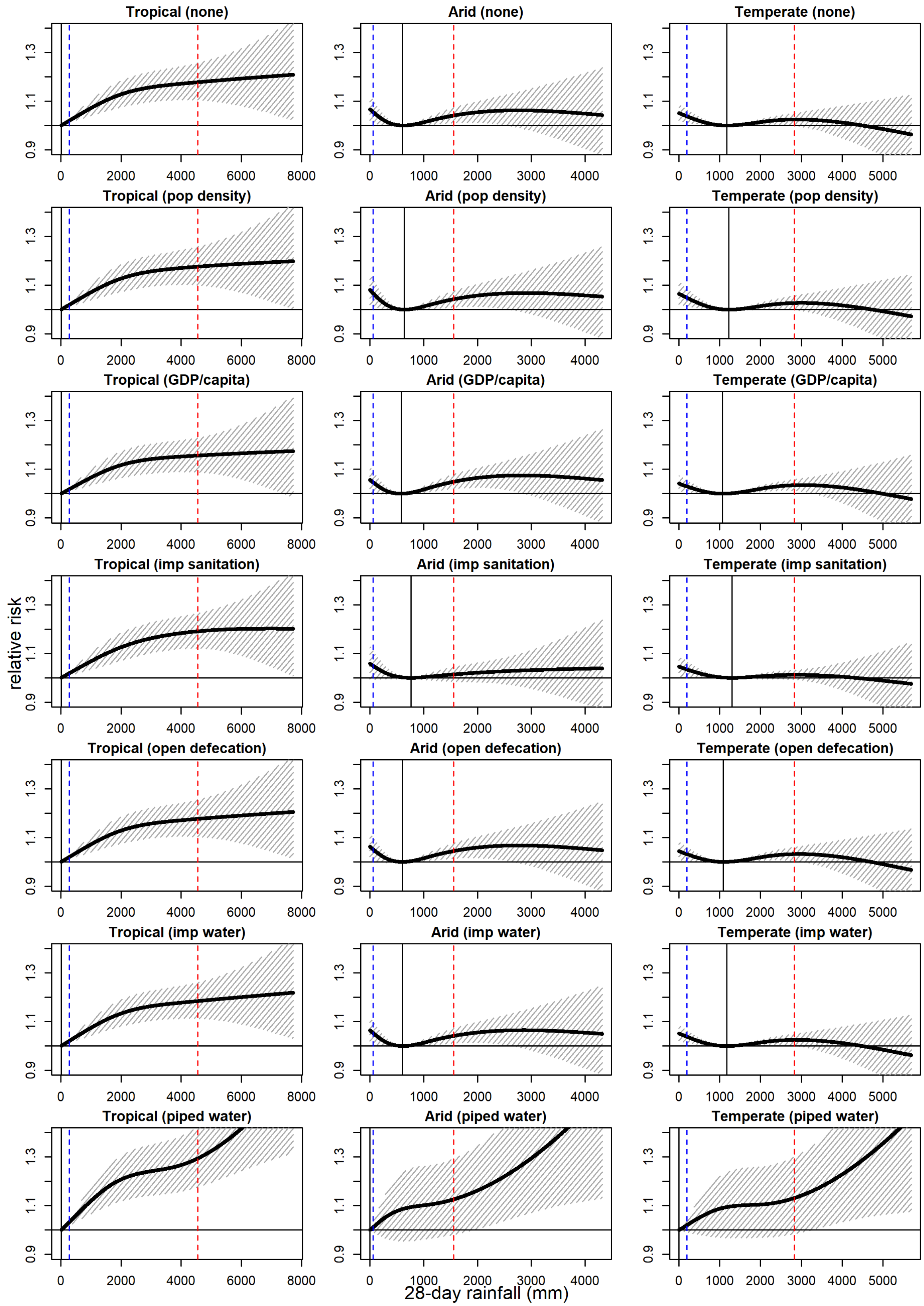

Figure 9. Sensitivity analysis of precipitation–diarrheal mortality associations by incorporating various meta-predictors

Black vertical lines are minimum risk precipitation. Blue and red broken lines are 5th percentile and 95th percentile of prior 28-day precipitation running sums. Black solid horizontal line is relative risk 1.00.

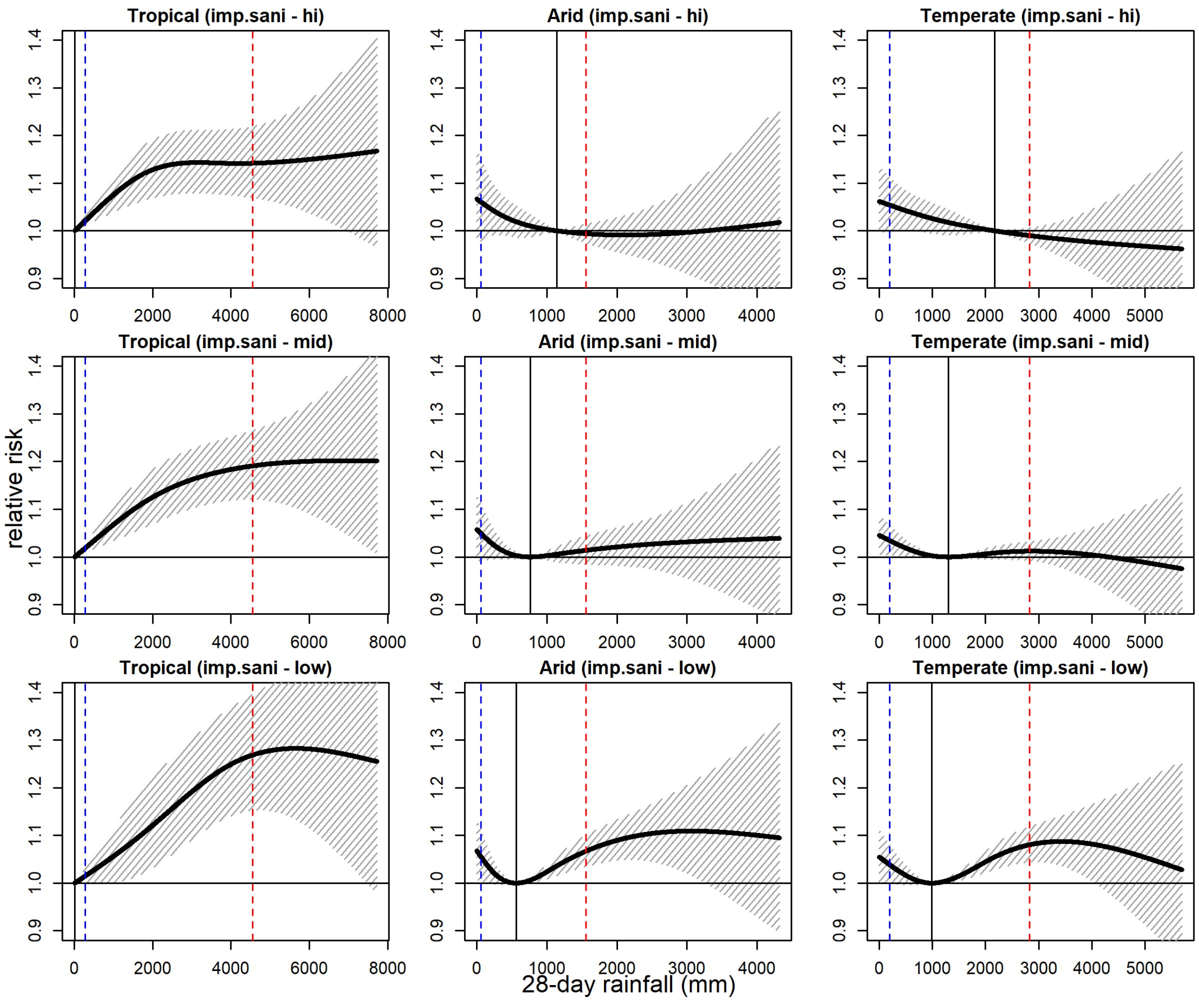

Figure 10. Sensitivity analysis of precipitation–diarrheal mortality associations by changing the percentage of improved sanitation coverage

"hi" refers to the 95th percentile of improved sanitation coverage at 98.7%, "mid" refers to the mean value of 78.7%, and "low" refers to the 5th percentile at 48.6%. Black vertical lines are minimum risk precipitation. Blue and red broken lines are 5th percentile and 95th percentile of prior 28-day precipitation running sums. Black solid horizontal line is relative risk 1.00.

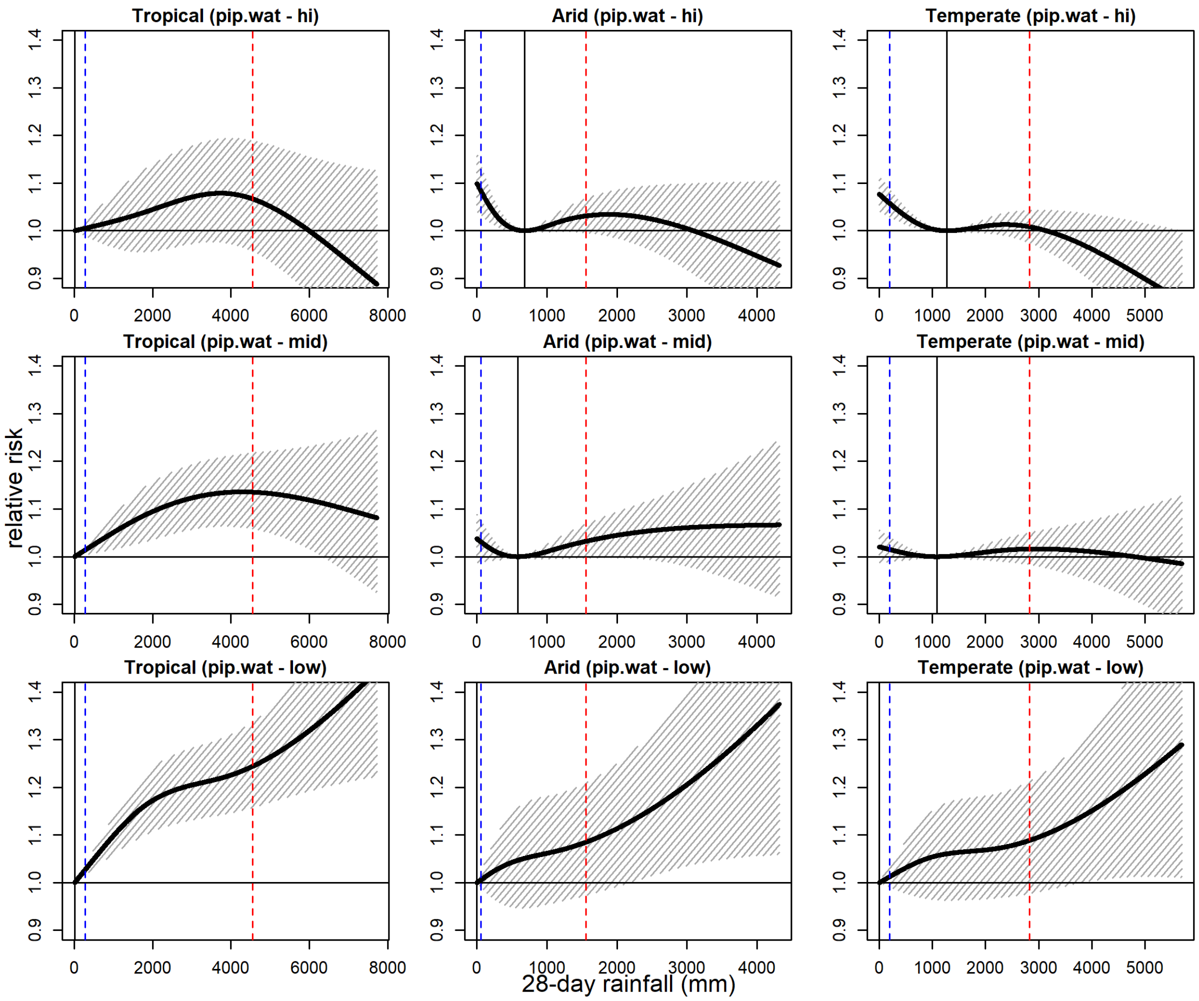

Figure 11. Sensitivity analysis of precipitation–diarrheal mortality associations by changing the percentage of piped water coverage

"hi" refers to the 95th percentile of piped water coverage at 96.9%, "mid" refers to the mean value of 70.2%, and "low" refers to the 5th percentile at 31.3%. Black vertical lines are minimum risk precipitation. Blue and red broken lines are 5th percentile and 95th percentile of prior 28-day precipitation running sums. Black solid horizontal line is relative risk 1.00.

REFERENCES

1. Munoz-Sabater J, Dutra E, Agusti-Panareda A, et al. ERA5-Land: a state-of-the-art global reanalysis dataset for land applications. *Earth System Science Data* 2021;**13**(9):4349-4383.
2. Beck HE, Zimmermann NE, McVicar TR, et al. Present and future Koppen-Geiger climate classification maps at 1-km resolution. *Scientific Data* 2018;**5**.
3. Peel MC, Finlayson BL, McMahon TA. Updated world map of the Koppen-Geiger climate classification. *Hydrology and Earth System Sciences* 2007;**11**(5):1633-1644.
4. Farrar DS, Awasthi S, Fadel SA, et al. Seasonal variation and etiologic inferences of childhood pneumonia and diarrhea mortality in India. *Elife* 2019;**8**.
5. Kummu M, Taka M, Guillaume JHA. Data Descriptor: Gridded global datasets for Gross Domestic Product and Human Development Index over 1990-2015. *Scientific Data* 2018;**5**.
6. Deshpande A, Miller-Petrie MK, Lindstedt PA, et al. Mapping geographical inequalities in access to drinking water and sanitation facilities in low-income and middle-income countries, 2000-17. *Lancet Global Health* 2020;**8**(9):E1162-E1185.
